# Microscopy of a Tooth. Osteo Dentine. Case No. 2

**Published:** 1871-02

**Authors:** S. P. Cutler


THE
### AMERICAN JOURNAL
OF
### DENTAL SCIENCE.

Vol. VII. THIRD SERIES--FEBRUARY, 1871. No. 10.

### ARTICLE I.

### *Microscopy of a Tooth. Osteo Dentine. Case No. 2.*

By S. P. Cutler, M. D., D. D. S.

A second lower molar tooth standing alone, of a young man 26 years old, and of a nervous, temperament, was extracted except the anterior fang which was left in the jaw. This tooth was decayed on the posterior approximal side, the cavity extending to the grinding surface, and reaching the nerve cavity, which had an ossified pulp nearly complete except over the broken fang, which was narrowed but had a live branch of nerve ; the main portion of cavity was completely ossified, the ossified pulp being perfectly white like the other dentine—this posterior fang was completely ossified to the apex. The ossification must have taken place before the decay quite reached the pulp cavity, from the fact that ti had extended into the secondary dentine to a limited extent, which was of a brown color darkened by the use of tobacco. The process must have first commenced just under the point of decay, and extended across and downwards into the fang,it being confined mainly to the pulp, as the narrowing of the pulp chamber was limited, the ossified pulp filling it completely, the line of demarcation being beautifully defined. What is most remarkable in this specimen is the bone corpuscles or cells, genuine lacunæ and canaliculi, in the fang portion as complete as in the cement, though not occupying the entire dentine, only around the outer layer of cells of the pulp, the inner portion presenting the usual appearance of secondary dentine under the microscope, only not so regular owing to a hurried condition of the process resulting, in some portions, of almost complete obliteration of nerve fibrils and what should have been secondary tubuli. In some regions, especially around the border of the pulp, were very large and extensive *lakes or lacunæ* large enough for a good sized microscopic whale to bask in with perfect freedom. These were in connection with the true bone cells also in the crown portion.

This is the second case of true bone corpuscles that I have met with in secondary dentine, and the first in a permanent tooth from hundreds of specimens. How many times others may have witnessed them I am unable to say, as they have not been published to my recollection. The bare report of such cases amounts to but little unaccompanied by theory or hypothesis; whether true or false they are better than none at all. My views of such cases are these: That when a tooth decays so as to remove a considerable portion of dentine there is necessarily removed a correspondiug amount of vital resistance in the pulp immediately under the decay removed, as the nerve fibrils so removed connect directly with the brain. Now in order that normal circulation should be kept up in any part there must be a normal amount of neural stimulus always in such part, otherwise stacis in the capillaries takes place and consequent infiltration into surrounding tissues of liquor sanguinis to a greater or less extent. In the case of a decayed tooth if there is no actual uneasiness felt in the tooth at all, there is beyond any doubt a weakened condition of pulp resistance, and in a corresponding manner the blood in the pulp, in part or all, circulates more languidly, thereby allowing more time for entrance of liquor sanguinis and consequently lime salts into pulp substance, which gradually hardens in the form of minute granules forming neuclei for further accumulation of lime salts, which may be regarded as the origin of the process, the pulp substance being a favorable matrix for ossification when nerve resistance has been overcome more or less,and the process being governed *paripassu*,by resistance or non-resistance. When the destruction of dentine has been considerable, and much irritation exists, the process may be hurried, as in the above named case, to such an extent that neural resistance is *nil*, when true bone corpuscles are formed on the same plan or principle of secondary bone formation, in cases of fractures or other injuries of bone, as the bone matrix is always of very low vitality, and vital resistance, otherwise ossification, could not take place under any circumstances

There is a law in bone formation as yet very imperfectly understood, we can simply recognise the process and not much more.

It would seem that there is a normal balance in the proportion of the animal and mineral constituents of teeth, favorable to normal healthy action and reaction, resisting for a long series of years any undue encroachments of the mineral elements upon the more vital, when unaffected by disease or decay. Any injury done to teeth, such as undue jarring by biting hard substances, absorption of alveolus, loosening from salivation or any other cause, undue collection of tartar around the teeth, and even constitutional disturbances, one and all may contribute to these secondary processes in an undue or abnormal manner. Wearing away by mastication, by the use of tobacco, and when other teeth are wanting to masticate upon, by longevity, one and all of the above cited cases tend to the same end or result, differing only in degree and time.

In that of longevity, where there has been no undue service, only normal mastication of food, there is simply a narrowing of tubular canals and pulp chamber, by deposit on inner walls, by regular integration of lime salts, preceeded no doubt by colloid matter to hold the salts until the entire interior of the tooth is more or less completely occluded, and its vitality ebbs *paripassu* with the integrating process. The modern "batter-whanging" on teeth with the mallet, or over forcible hand-pressure in filling, tend to favor this, as ossifications are much more frequently met now than twenty years ago. There are certain temperaments, such as the nervous, more prone to secondary ossific formations, than others, such as the bilious, which has more resisting vital force. The lymphatico-nervous might be regarded as the most susceptible, while the bilio-sanguine is the most resistant of mixed temperaments. When an aching tooth presents itself, we are not always sure that there is ossification existing, especially when the sensibility will not allow of careful examination before making applications to destroy the pulp; prior examination will not always reveal the true condition. In cases of spicular nodules in the pulp, we cannot determine the fact before removal of the pulp, though in such cases where the deposit is not extensive there is not great difficulty in the removal of such nerve by the branch, and where there is sufficient opening of nerve cavity they may be destroyed by arsenic. In the above cited case we have two bone corpuscles or cells in certain regions, secondary dentine in others, showing nerve plexuses and tubuli, in other regions extensive caverns, others again uniform homogenous structure, where all traces of organic tissue structure are obliterated, clearly indicating the hurried condition of the process. When bone corpuscles prevail, we necessarily conclude that all vital resistance has succumbed to the ravages of mineral metamorphosis; in other regions nerve resistance determined the process and resulted in secondary dentine. I have recently met with ossified pulp in deciduous teeth, where the process of absorption had been perfectly normal in all respects. I have a specimen of molar showing true secondary dentine, where absorption had extended into the pulp chamber by the way of crown, others more or less absorbed away by normal process, unaffected by decay in some instances, others decayed more or less. All such cases when carefully studied, tend to shed light on our limited stock of histo-pathological knowledge, which I am sorry to say is sadly neglected by our profession. The practical issue of the case is, whether or not there can be any means desired by which ossification can be made to take place under the region of extensive decay, so as to protect the pulp in that direction from the shocks of fillings of any description, differing somewhat among themselves, which are either favorable to this process or to the death of pulp, as the pulp cannot long remain *status-quo* in such cases. Even if nodular dentine should be the result, unless the process should be to much hurried, the same conservative end will be accomplished and more or less of the pulp remain vital, sufficient to save the tooth from *articulo mortis.* The question is how shall we treat and manage teeth in order to accomplish this desirable end. Duty is knocking loudly at our doors for response, we owe this debt to humanity, to ourselves and the rest of mankind.

---

